# Response of CH_4_ and N_2_O Emissions and Wheat Yields to Tillage Method Changes in the North China Plain

**DOI:** 10.1371/journal.pone.0051206

**Published:** 2012-12-06

**Authors:** Shenzhong Tian, Tangyuan Ning, Hongxiang Zhao, Bingwen Wang, Na Li, Huifang Han, Zengjia Li, Shuyun Chi

**Affiliations:** 1 State Key Laboratory of Crop Biology, Shandong Key Laboratory of Crop Biology, Shandong Agricultural University, Taian, Shandong PR, China; 2 College of Mechanical and Electronic Engineering, Shandong Agricultural University, Taian, Shandong PR, China; DOE Pacific Northwest National Laboratory, United States of America

## Abstract

The objective of this study was to quantify soil methane (CH_4_) and nitrous oxide (N_2_O) emissions when converting from minimum and no-tillage systems to subsoiling (tilled soil to a depth of 40 cm to 45 cm) in the North China Plain. The relationships between CH_4_ and N_2_O flux and soil temperature, moisture, NH_4_^+^-N, organic carbon (SOC) and pH were investigated over 18 months using a split-plot design. The soil absorption of CH_4_ appeared to increase after conversion from no-tillage (NT) to subsoiling (NTS), from harrow tillage (HT) to subsoiling (HTS) and from rotary tillage (RT) to subsoiling (RTS). N_2_O emissions also increased after conversion. Furthermore, after conversion to subsoiling, the combined global warming potential (GWP) of CH_4_ and N_2_O increased by approximately 0.05 kg CO_2_ ha^−1^ for HTS, 0.02 kg CO_2_ ha^−1^ for RTS and 0.23 kg CO_2_ ha^−1^ for NTS. Soil temperature, moisture, SOC, NH_4_^+^-N and pH also changed after conversion to subsoiling. These changes were correlated with CH_4_ uptake and N_2_O emissions. However, there was no significant correlation between N_2_O emissions and soil temperature in this study. The grain yields of wheat improved after conversion to subsoiling. Under HTS, RTS and NTS, the average grain yield was elevated by approximately 42.5%, 27.8% and 60.3% respectively. Our findings indicate that RTS and HTS would be ideal rotation tillage systems to balance GWP decreases and grain yield improvements in the North China Plain region.

## Introduction

CH_4_ and N_2_O play a key role in global climate change [1]. The emission of gas from disturbed soils is an especially important contributory factor to global change [2]. N_2_O is emitted from disturbed soil, whereas CH_4_ is normally oxidized by aerobic soils, making them sinks for atmospheric CH_4_ in dry farmland systems [3]. According to estimates of the IPCC [4], CH_4_ and N_2_O from agricultural sources account for 50% and 60% of total emissions, respectively. Therefore, it is critical to reduce emissions of greenhouse gases (GHG) from agricultural sources. Many studies have reported that soil tillage has significant effects on CH_4_ and N_2_O emissions from farmland because the production, consumption and transport of CH_4_ and N_2_O in soil are strongly influenced by tillage methods [5]–[8].

The North China Plain is one of the most important grain production regions of China. Harrow tillage (HT), rotary tillage (RT) and no-tillage (NT) are frequently used conservation tillage methods in this region because they not only improve crop yield but also enhance the utilization efficiency of soil moisture and nutrients [8]–[12]. However, successive years of shallow tillage (10–20 cm) exacerbate the risk of subsoil compaction, which not only leads to the hardening of soil tillage layers and an increase in soil bulk density, but also reduced crop root proliferation, limited water and nutrient availability and reduced crop yield [13]. Subsoiling is an effective method that is used to break up the compacted hardpan layer every 2 or 4 years in HT, RT or NT systems [14], [15]. Subsoiling significantly increases soil water content and temperature and decreases soil bulk density as well [16], [17]. These rotation tillage systems are currently utilized in the North China Plain. Soil moisture and temperature are two factors controlling CH_4_ and N_2_O emissions [18]–[22]. In addition, CH_4_ and N_2_O emissions are normally associated with N application (as fertilizer) under wet conditions [23].

Collectively, reasonable soil tillage methods may reduce GHG emissions and may be important for developing sustainable agricultural practices [24]. However, it is unclear how conversion to subsoiling would affect CH_4_ and N_2_O emissions and whether subsoiling increases or reduces GHG emissions and the GWP of these agricultural techniques. In addition, there is little information on the soil factors affecting CH_4_ and N_2_O emissions after conversion to subsoiling in the North China Plain. The aim of this study was to determine whether conversion to subsoiling can reduce CH_4_ and N_2_O emissions.

## Materials and Methods

### Ethics Statement

The research station of this study is a department of Shandong Agricultural University. This study was approved by State Key Laboratory of Crop Biology, Shandong Key Laboratory of Crop Biology, Shandong Agricultural University.

### Study Site

**Figure 1:**
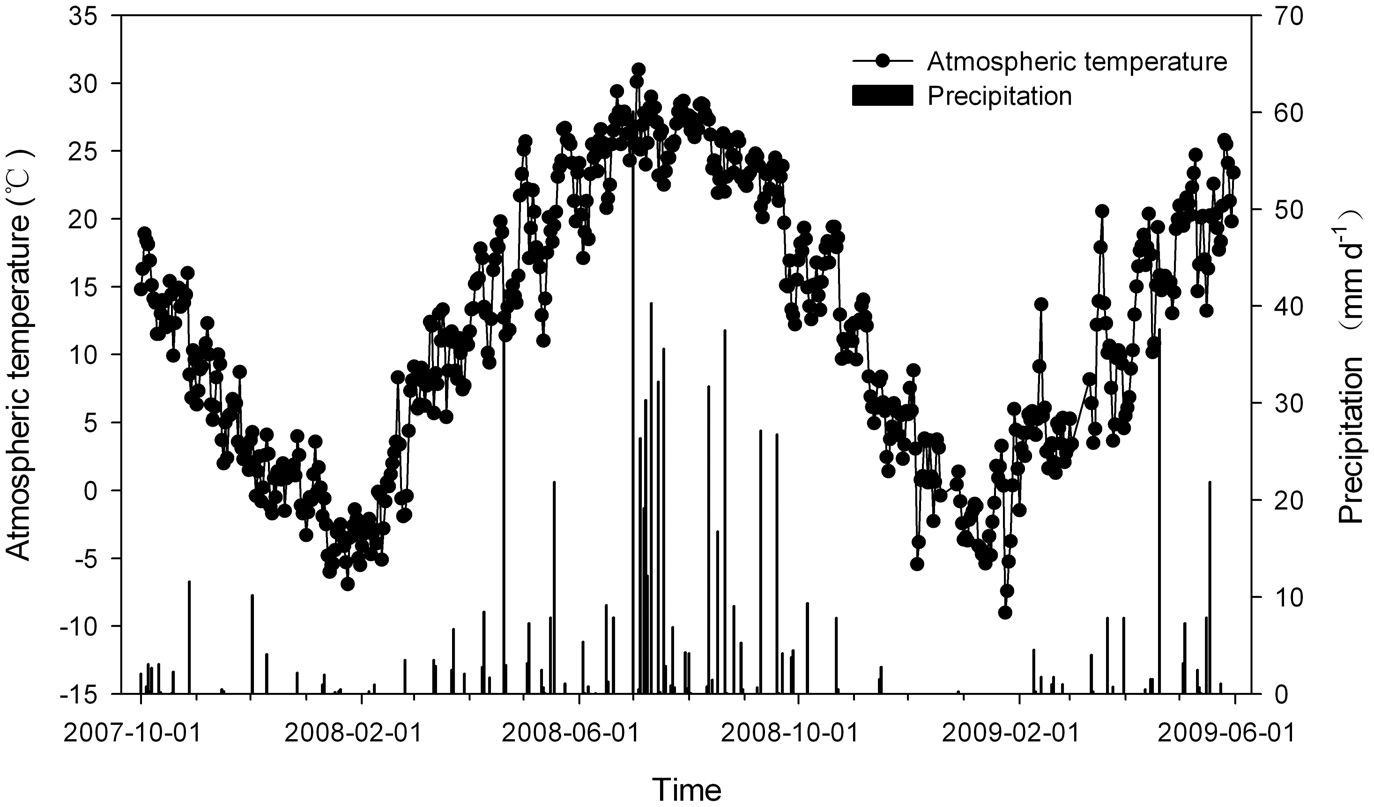
The atmospheric temperature and precipitation at the experiment site. The data were collected by the agricultural meteorological station approximately 500 m from the experiment field.

The study was conducted at Tai’an (Northern China, 36°09′N, 117°09′E), which is characteristic of the North China Plain. The average annual precipitation is 786.3 mm, and the average annual temperature is 13.6°C, with the minimum (−1.5°C) and maximum (27.5°C) monthly temperatures in January and July, respectively. The annual frost-free period is approximately 170–220 days in duration, and the annual sunlight time is 2462.3 hours. The soil is loam with 40% sand, 44% silt and 16% clay. The characteristics of the surface soil (0–20 cm) were measured as follows: pH 6.2; soil bulk density 1.43 g cm^−3^; soil organic matter 1.36%; soil total nitrogen 0.13%; and soil total phosphorous 0.13%. The meteorological data during the experiment are shown in Figure 1.

### Experimental Design

The experiment was designed as HT, RT and NT farming methods that started in 2004. In 2008, each plot was bisected, with one half maintained using the original tillage method as the control and the other half converted to subsoiling, resulting in six treatment plots: HT and HT conversion to subsoiling (HTS); RT and RT conversion to subsoiling (RTS); and NT and NT conversion to subsoiling (NTS) in a split-plot design with three replicates. Each replicate was 35 m long and 4 m wide. After maize was harvested in each plot, straw was returned to the soil by one of the six following tillage operations:

HT - disking with a disc harrow to a depth of 12 cm to 15 cm,

RT - rototiller plowing to a depth of 10 cm to 15 cm,

NT - no tillage,

HTS, RTS, and NTS - plowed using a vibrating sub-soil shovel to a depth of 40 cm to 45 cm,

The experimental site was cropped with a rotation of winter wheat (*Triticum aestivum* Linn.) and maize (*Zea mays L*.). The wheat was sown in mid-October immediately after tilling the soil and was harvested at the beginning of June the following year. The maize was sown directly after the wheat harvest and was harvested in early October. During the wheat growth period, fertilizer was used at a rate of 225 kg N ha^−1^, 150 kg ha^−1^ P_2_O_5_ and 105 kg ha^−1^ K_2_O, and 100 kg N ha^−1^ was used as topdressing in the jointing stage with 160 mm of irrigation water. During the maize growth period, 120 kg N ha^−1^, 120 kg ha^−1^ P_2_O_5_ and 100 kg ha^−1^ K_2_O were used as a base fertilizer, and 120 kg N ha^−1^ was used as topdressing in the jointing stage.

### CH_4_ and N_2_O Sampling and Measurements

CH_4_ and N_2_O content was measured using the static chamber-gas chromatography method [25]. The duration of gas sample collection was based on the diurnal variations in this region: the collection of CH_4_ occurred from 9∶00 a.m. to 10∶00 a.m., and N_2_O was collected between 9∶00 a.m. and 12∶00 p.m. from October 10, 2007, to May 19, 2009 at approximately 1-month intervals [26]. Both CH_4_ and N_2_O were sampled at 5 minutes, 20 minutes and 35 minutes after chamber closing. Simultaneously, the atmospheric temperature, the temperature in the static chamber, the land surface temperature and the soil temperature at a depth of 5 cm were determined after collecting samples.

The samples were measured using a Shimadzu GC-2010 gas chromatograph. CH_4_ was measured using a flame ionization detector with a stainless steel chromatography column packed with a 5A molecular sieve (2 m long); the carrier gas was N_2_. The temperatures of the column, injector and detector were 80°C, 100°C and 200°C, respectively. The total flow of the carrier gas was 30 ml min^−1^, the H_2_ flow was 40 ml min^−1^, and the airflow was 400 ml min^−1^. N_2_O was measured using an electron capture detector with a Porapak-Q chromatography column (4 m long); the carrier gas was also N_2_. The temperatures of the column, injector and detector were 45°C, 100°C and 300°C, respectively. The total flow of the carrier gas was 40 ml min^−1^, and the tail-blowing flow was 40 ml min^−1^. The gas fluctuations were calculated by the gas concentration change in time per unit area.

Emission changes in CH_4_ and N_2_O were calculated using the following formula [25]:
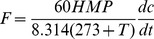
where *F* is the change in gas emission or uptake (µg·m^−2^·h^−1^); 60 is the conversion coefficient of minutes and hours; H is the height (m); M is the molar mass of gas (g·mol^−1^); P is the atmospheric pressure (Pa); 8.314 is the Ideal Gas Constant (J mol^−1^ K^−1^); T is the average temperature in the static chamber (°C); and *dc/dt* is the line slope of the gas concentration change over time.

### GWP of CH_4_ and N_2_O

The global warming potentials (GWP) were determined by measuring CH_4_ and N_2_O emissions. The GWP of CH_4_ and N_2_O are 25 and 298 times higher, respectively, than that of CO_2_ (the GWP of CO_2_ is 1) [27] and are calculated as follows:
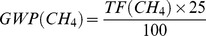


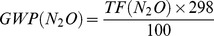
where *GWP(CH_4_)* is the GWP of CH_4_ (kg CO_2_ ha^−1^); *TF(CH_4_)* is the total uptake of CH_4_ (kg CO_2_ ha^−1^ a^−1^); 25 is the GWP coefficient of CH_4_; 100 is the time scale of climate change (a); *GWP(N_2_O)* is the GWP of N_2_O (kg CO_2_ ha^−1^); *TF(N_2_O)* is the total emission of N_2_O (kg CO_2_ ha^−1^ a^−1^); and 298 is the GWP coefficient of N_2_O.

### Soil Factor Measurements

The meteorological data during the experiment were obtained from an agricultural weather station in the experimental area. To evaluate the relation between soil temperature and moisture and CH_4_ and N_2_O emissions, we measured soil temperature at a depth of 5 cm and the soil moisture in the 0–20 cm soil layers simultaneously using a soil temperature, moisture and electric conductivity instrument (WET brand, made in the UK) as the temperature and moisture data collection tool. The soil samples were collected using a soil sampler with five replicates in each different tillage treatment and were dried and triturated after mixing. This sample was used to determine the SOC, NH_4_^+^-N and pH using the Potassium Dichromate Heating Method, the UV Colorimetric Method and the Potentiometry Method, respectively [28].

### Grain Yield

The grain yield of winter wheat was sampled from the 1.5 m× 6 m portion in the central area of each plot.

### Statistical Analyses

The data were analyzed using analyses of variance and the SPSS 17.0 Statistical Analysis System and were mapped using Sigma Plot 10.0. The mean standard deviation and least significant difference were calculated for comparison of the treatment means.

## Results

### CH_4_ and N_2_O

**Figure 2:**
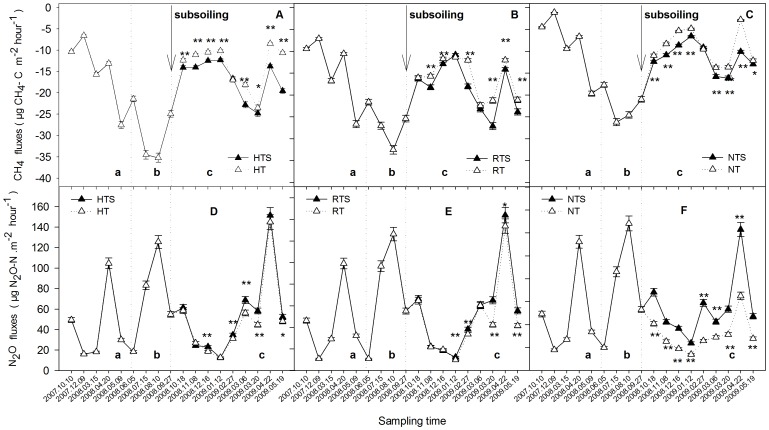
A to C CH_4_ flux variations of H, R, and N after subsoiling in different periods; D to F N_2_O flux variations of H, R, and N after subsoiling in different periods. **a** in Fig. 2 is the wheat growth stage of 2007 to 2008; **b** is the maize growth stage of 2008 to 2009; c is the wheat growth stage of 2008 to 2009. Arrows indicate time of subsoiling. Dotted lines distinguish the growth period of wheat and maize. * indicates *P*<0.05 and **indicates *P*<0.01 between subsoiling and the control.

Differences in CH_4_ flux were observed when converting from HT to HTS, from RT to RTS and from NT to NTS (Figs. 2 A to C). The soil absorption of CH_4_ increased in different periods after conversion to subsoiling compared with the control. The soil absorption of CH_4_ increased from 13.53 µg·m^−2^·h^−1^ under HT to 16.72 µg·m^−2^·h^−1^ under HTS, from 15.59 µg·m^−2^·h^−1^ under RT to 18.20 µg·m^−2^·h^−1^ under RTS and from 9.01 µg·m^−2^·h^−1^ under NT to 11.36 µg·m^−2^·h^−1^ under NTS, respectively. However, N_2_O emission also increased after subsoiling (Fig. 2 D to F), which increased from 49.07 µg·m^−2^·h^−1^ under HT to 54.05 µg·m^−2^·h^−1^ under HTS and from 47.49 µg·m^−2^·h^−1^ under RT to 53.60 µg·m^−2^·h^−1^ under RTS. Compared with the above two treatments, however, the N_2_O emissions from the soil after conversion to NTS increased significantly, from 30.92 µg·m^−2^·h^−1^ under NT to 55.15 µg·m^−2^·h^−1^ under NTS.

### GWP of CH_4_ and N_2_O

CH_4_ uptake increased under HTS, RTS and NTS; consequently, the GWP of CH_4_ decreased using these tilling methods compared with HT, RT and NT. However, the GWP of N_2_O increased under HTS, RTS and NTS (Table 1). Overall, therefore, the GWPs of the CH_4_ and N_2_O emissions taken together increased from 0.32 kg CO_2_ ha^−1^ under HT to 0.37 kg CO_2_ ha^−1^ under HTS, from 0.37 kg CO_2_ ha^−1^ under RT to 0.39 kg CO_2_ ha^−1^ under RTS and from 0.26 kg CO_2_ ha^−1^ under NT to 0.49 kg CO_2_ ha^−1^ under NTS, respectively.

**Table 1:** GWP and total changes in CH_4_ and N_2_O after subsoiling (2008.10∼2009.05).

Treatments	HT	HTS	RT	RTS	NT	NTS
CH_4_ total emission (kg·ha^−1^)	−0.73	−0.84	−0.64	−0.78	−0.39	−0.52
GWP of CH_4_ (kgCO_2_ ·ha^−1^)	−0.17	−0.19	−0.15	−0.18	−0.09	−0.12
N_2_O total emission (kg·ha^−1^)	2.14	2.42	2.26	2.46	1.46	2.67
GWP of N_2_O (kgCO_2_ ·ha^−1^)	0.49	0.56	0.52	0.57	0.35	0.61
Total emissions of CH_4_ and N_2_O (kg·ha^−1^)	1.41	1.58	1.62	1.68	1.07	2.15
GWP of CH_4_ and N_2_O (kgCO_2_ ha^−1^)	0.32	0.37	0.37	0.39	0.26	0.49
Increased emissions after conversion (kg·ha^−1^)	–	0.17	–	0.06	–	1.08
Increased GWP after conversion(kgCO_2_ ·ha^−1^)	–	0.05	–	0.02	–	0.23

**Total emissions of CH_4_ and N_2_O (kg·ha^−1^),** N_2_O total emission flux added CH_4_ total emission flux; **GWP of CH_4_ and N_2_O (kgCO_2_·ha^−1^),** GWP of N_2_O added GWP of CH_4_; **Increased emissions after conversion (kg·ha^−1^),** difference of total emission of CH_4_ and N_2_O before and after conversion; **Increased GWP after conversion (kgCO_2_·ha^−1^),** difference of GWP of CH_4_ and N_2_O before and after conversion.

### Correlation Analysis between CH_4_ and N_2_O and Soil Factors

Soil temperature significantly affected the CH_4_ uptake in soils, especially in lower (i.e., December, R^2^ = 0.7314, *P*<0.01; January, R^2^ = 0.6490, *P*<0.01; February, R^2^ = 0.6597, *P*<0.01) or higher (i.e., May, R^2^ = 0.8870, *P*<0.01) temperatures (*P*<0.01) (Table 2). At other sampling times, however, temperature did not affect on CH_4_ uptake, and soil moisture became a main influencing factor on the absorption of CH_4_ by the soils, especially in wet soil, such as after rain (R^2^ = 0.5154, *P*<0.05) and irrigation (R^2^ = 0.5154, *P*<0.05), when CH_4_ absorption was significantly limited (R^2^ = 0.5429, *P*<0.05). Higher soil moisture generally promoted the emission of N_2_O (R^2^ = 0.6735, *P*<0.01), but there was no obvious correlation between soil temperature and N_2_O emissions.

**Table 2:** Correlation analysis between changes in CH_4_ and N_2_O with soil temperature and soil moisture per sampling time.

Sampling time	Soil temperature				Soil moisture			
	CH_4_		N_2_O		CH_4_		N_2_O	
	*R^2^*	*n*	*R^2^*	*n*	*R^2^*	*n*	*R^2^*	*n*
2008.10.18	0.6020*	3	0.3832	3	0.5429*	3	0.1020	3
2008.11.08	0.6180*	3	0.0377	3	0.2945	3	0.1241	3
2008.12.16	0.7314**	3	0.0087	3	0.0085	3	0.5142*	3
2009.01.12	0.6490**	3	0.0723	3	0.2988	3	0.5200*	3
2009.02.27	0.6597**	3	0.3053	3	0.5370*	3	0.0914	3
2009.03.06	0.3824	3	0.1461	3	0.0417	3	0.0005	3
2009.03.20	0.2876	3	0.0257	3	0.4966*	3	0.6132*	3
2009.04.22	0.4476*	3	0.3044	3	0.5154*	3	0.6735**	3
2009.05.19	0.8870**	3	0.0503	3	0.4593*	3	0.5027*	3

* *P*<0.05,

** *P*<0.01.

**Figure 3:**
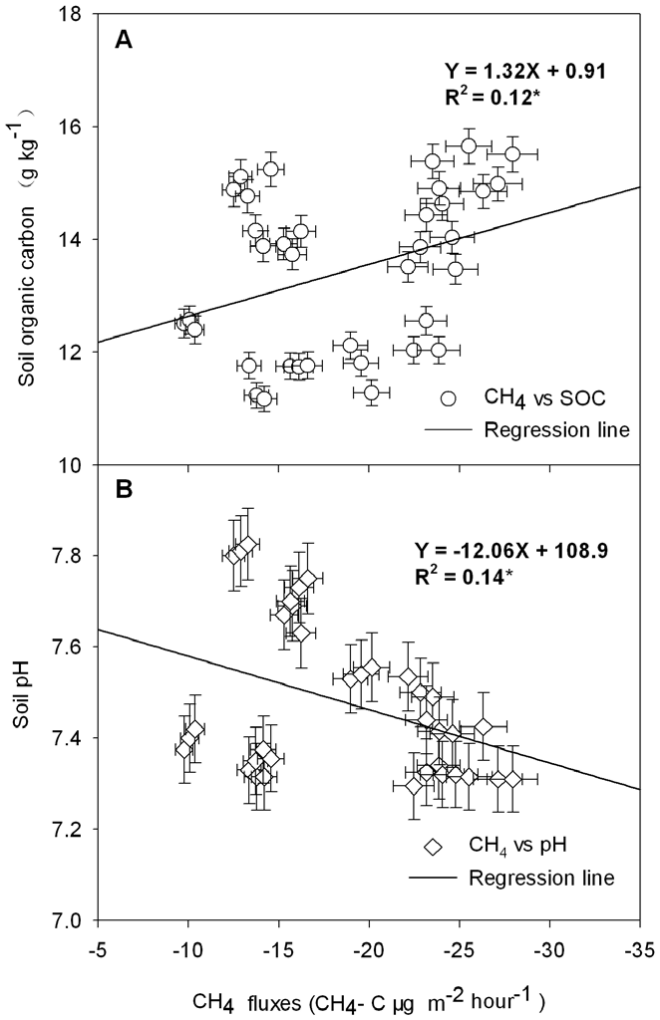
A Linear regression between the CH_4_ uptake fluxes and SOC, B Linear regression between the CH_4_ uptake fluxes and soil pH. Arrows indicate the regression equation between the CH_4_ uptake fluxes and soil organic carbon, soil pH. *indicates *P*<0.05.

In this study, SOC was also correlated with greater CH_4_ uptake (R^2^ = 0.12, *P*<0.05) (Fig. 3 A), whereas higher soil pH limited its absorption in the soil (R^2^ = 0.14, *P*<0.05) (Fig. 3 B).

**Figure 4:**
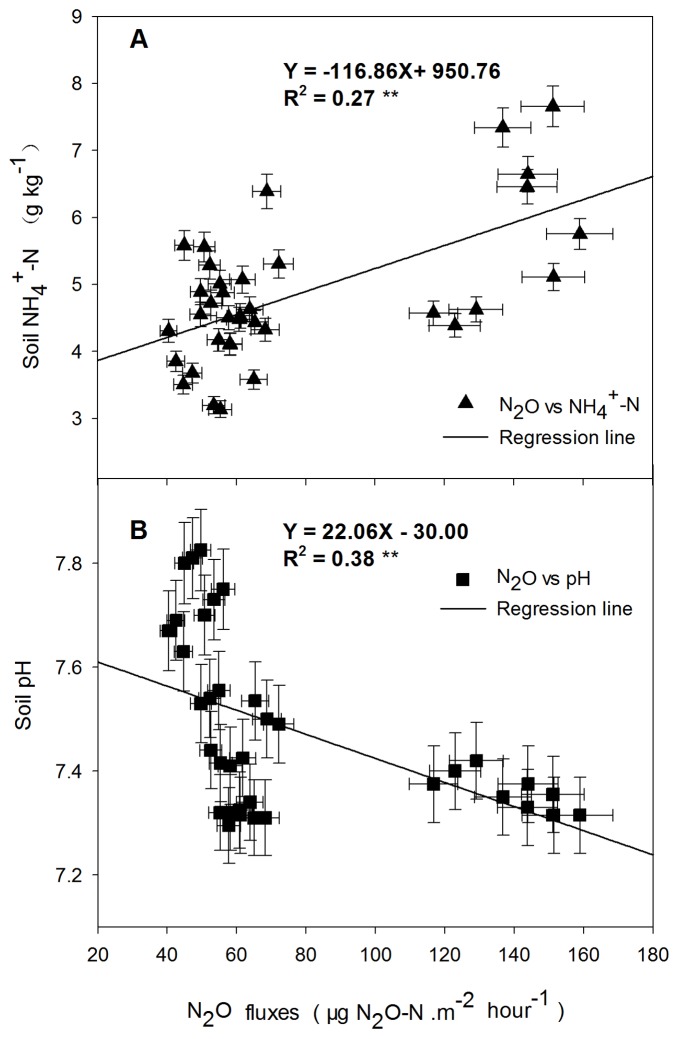
A Linear regression between the N_2_O emission fluxes and soil NH_4_^+^-N, B Linear regression between the N_2_O emission fluxes and soil pH. Arrows indicate the regression equation between the N_2_O emission fluxes and soil NH_4_^+^-N, soil pH. **indicates *P*<0.01.

The emission of N_2_O was correlated with higher soil NH_4_^+^-N content (R^2^ = 0.27, *P*<0.01) (Fig. 4 A), while, similar to CH_4_, a higher pH in soil strongly limited the emission of N_2_O (R^2^ = 0.38, *P*<0.01) (Fig. 4 B).

### Variation of Soil Factors

**Figure 5:**
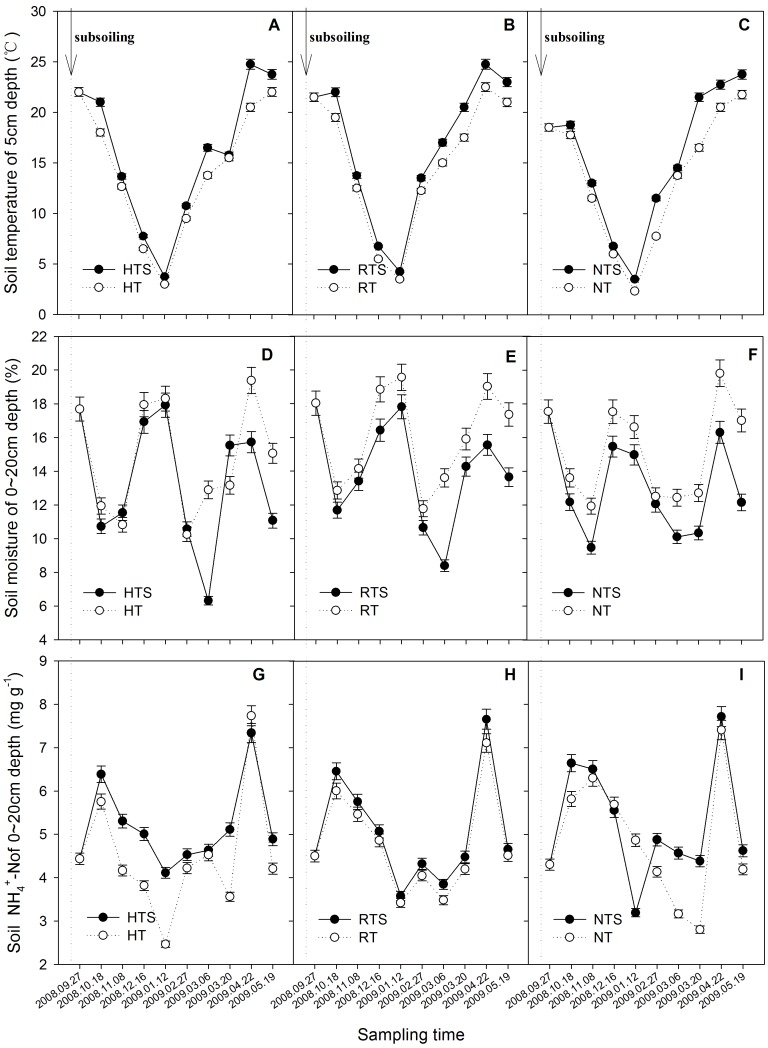
A to C Variation of Soil temperature at a 5 cm depth (°C) after subsoiling; D to F Variation of Soil water content at a 0∼20 cm depth (%) after subsoiling; G to I Variation of Soil NH_4_^+^-N at a 0∼20 cm depth (mg·kg^−1^) after subsoiling. Arrows and the dotted line indicate time of subsoiling.

The soil factors under HTS, RTS and NTS changed after subsoiling. The soil temperature at a depth of 5 cm rose under HTS, RTS and NTS compared with the temperatures under HT, RT and NT (Fig. 5 A to C). Soil temperature variations followed atmospheric temperature changes, but the average soil temperature during sampling period increased from 13.5°C under HT to 15.3°C under HTS, from 14.4°C under RT to 16.2°C under RTS and from 13.1°C under NT to 15.1°C under NTS, respectively. However, soil moisture decreased in the soil at 0–20 cm when converting to subsoiling that in the order of RTS>HTS>NTS (Fig. 5 D to F). The most obvious decrease, by 15.74%, occurred under the NTS treatment, while HTS and RTS decreased by 10.34% and 14.85%, respectively. The soil NH_4_^+^-N content increased with subsoiling that was NTS>HTS>RTS. Moreover, two peaks occurring on October 18, 2008, and April 22, 2009 (Fig. 5 G to I), due to the application of nitrogenous base fertilizer and topdressing fertilizer.

The CH_4_ uptake and N_2_O emission were correlated with the content of soil pH and SOC (Table 3). The pH value decreased after conversions, but with the pH under the NTS treatment being higher than that of the HTS and RTS treatments not only at 0∼10 cm but also at 10∼20 cm. Conversely, SOC content increased under HTS, RTS and NTS, with the highest values was under RTS, followed by NTS and then HTS. SOC was higher in the soil at 0–10 cm than at 10–20 cm.

**Table 3:** Soil pH and SOC variations after conversion to subsoiling.

Treatments		pH						SOC					
		HT	HTS	RT	RTS	NT	NTS	HT	HTS	RT	RTS	NT	NTS
0∼10 cm	(i)	7.37^c^	7.33^d^	7.25^e^	7.21^f^	7.72^a^	7.66^b^	8.62^f^	9.45^e^	9.69^d^	11.47^b^	11.79^a^	10.32^c^
	(ii)	7.25^d^	7.21^e^	7.27^c^	7.25^d^	7.69^a^	7.62^b^	10.77^d^	12.25^a^	9.82^f^	10.21^e^	11.68^c^	11.93^b^
	(iii)	7.25^e^	7.23^f^	7.38^a^	7.34^c^	7.37^b^	7.31^d^	11.43^d^	12.58^b^	12.07^c^	13.11^a^	10.13^e^	9.75^f^
	(iv)	7.44^cd^	7.42^d^	7.45^c^	7.40^e^	7.86^a^	7.82^b^	9.01^f^	9.39^e^	10.83^b^	12.42^a^	10.57^c^	10.49^d^
10∼20 cm	(i)	7.71^c^	7.67^d^	7.52^e^	7.46^f^	7.77^a^	7.75^b^	5.93^f^	6.29^e^	9.10^b^	9.44^a^	8.09^d^	8.34^c^
	(ii)	7.46^c^	7.43^d^	7.36^e^	7.35^f^	7.85^a^	7.83^b^	9.22^f^	9.97^d^	9.45^e^	10.07^c^	11.35^b^	11.77^a^
	(iii)	7.44^c^	7.40^d^	7.39^e^	7.37^f^	7.56^a^	7.52^b^	9.76^f^	10.62^c^	10.11^e^	10.40^d^	10.88^b^	11.76^a^
	(iv)	7.71^c^	7.68^d^	7.43^e^	7.43^e^	7.83^a^	7.81^b^	7.63^f^	9.90^a^	8.26^d^	9.55^b^	8.31^c^	7.84^e^

Different small letter means *P*<0.01; (i), (ii), (iv) and (iii) means time of sample collection in 2008.10.18, 2009.03.17, 2009.04.20 and 2009.05.19 respectively.

**Table 4:** The wheat yield variations of HT, RT and NT after subsoiling from 2008–2010.

Treatments	Number of spikes(10^4^·ha^−1^)	Grains per ear	1000-grain weight(g)	Grain yield(kg·ha^−1^)	Increased(kg·ha^−1^)
2008–2009					
HT	646.50^bc^	30.05^bc^	33.79^b^	5582.83^b^	
HTS	683.50^a^	34.45^a^	34.31^b^	6866.55^a^	+1283.72
RT	655.00^b^	31.45^b^	33.94^b^	5937.20^b^	
RTS	637.50^c^	35.00^a^	36.83^a^	6985.20^a^	+1048.00
NT	583.00^d^	28.60^c^	32.40^c^	4595.87^c^	
NTS	688.50^a^	34.70^a^	33.96^b^	6895.06^a^	+2299.19
2009–2010					
HT	644.67^e^	30.93^e^	33.73^d^	5716.53^e^	
HTS	741.00^b^	38.59^a^	37.70^a^	9161.94^a^	+3548.77
RT	705.00^c^	31.68^d^	32.47^f^	6164.83^d^	
RTS	754.67^a^	35.78^c^	36.77^b^	8439.35^b^	+2342.76
NT	601.67^f^	28.02^f^	32.70^e^	4685.80^f^	
NTS	682.00^d^	37.72^b^	36.13^c^	7898.86^c^	+3309.46

Different small letter means *P*<0.05.

### Grain Yield

The highest wheat yields under RT were 5937.20 kg ha**^−^** ^1^ in 2009 and 6164.83 kg ha**^−^** ^1^ in 2010, which were only 3.8% greater than those under HT and NT (Table 4). However, the wheat yields under HTS, RTS and NTS improved significantly (*P*<0.01) than the control, not only in 2009 but also in 2010. The average yield of the two years increased by approximately 2416.25 kg ha**^−^** ^1^, 1695.38 kg ha**^−^** ^1^and 2804.33 kg ha**^−^** ^1^ with subsoiling compared with that under HT, RT and NT, respectively. The increases of average yield were not only related to the number of spikes, which increased by 59×10^4^ ha**^−^** ^1^ after conversions as determined by the average of the three conversion treatments, but were also correlated with the grains per ear and 1000-grain weight, which increased by an average of 6.0 grains and 2.8 g, respectively.

## Discussion

### Effect of Conversion to Subsoiling on CH_4_ Uptake and N_2_O Emissions

Long periods of shallow or no-tillage have resulted in an increase in soil bulk density and compacted hardpan in this region, especially in the subsoil [29], [30], while subsoiling changed the soil structure, allowing increased gas diffusion in the soil. In this study, soils under HT conversion to HTS, RT conversion to RTS and NT conversion to NTS increased CH_4_ absorption and strengthened the sink capacity of the soils (Fig. 2 A to C); however, these conversions also promoted the emission of N_2_O (Fig. 2 D to F). This increase may be due to changes in soil conditions as a result of conversion to tillage (Fig. 5). For example, the increase in CH_4_ absorption after conversion was mainly correlated with soil temperature, soil moisture, soil pH and SOC content according to the correlation analysis (Fig. 3 and Table 2), which is consistent with some previous studies [31]–[33]. A higher temperature and greater SOC may be advantageous to increasing the amount of CH_4_ absorbed by the soil (Table 2, Fig. 3A) [34], [35]. However, soil moisture and pH were two limiting factors in our study (Table 2, Fig. 3B) that had negative effects on CH_4_ absorption in the soils [36].

At the same time, subsoiling would reduce subsoil compaction, and some have found improved permeability of soil to increased soil methane sinks [37] and higher bulk density to limit gas diffusion from the soil to the atmosphere, prolonging methane transfer pathways and thereby reducing CH_4_ and O_2_ diffusion between the soil and the atmosphere [38]. Sometimes, although increased soil tillage may slightly decrease CH_4_ uptake [39], this effect is small and can be largely ignored [6], [40].

The conditions for the aeration of the soil profile were reduced after irrigation [41], [42] that increases emissions of the greenhouse gas N_2_O through denitrification in farmland [22], the N_2_O emission peaks also coincided with higher moisture and NH_4_^+^-N content in this study (Fig. 2 D to F, Table 2, Fig. 4A), the emissions of N_2_O were significantly affected by soil moisture and NH_4_^+^-N content in each treatment. Some studies have indicated that there is a significant linear relationship between N_2_O emissions and soil moisture and nitrogenous fertilizer [21], [22]. In addition, there was no significant correlation between N_2_O emission and soil temperature in this study, and similar results were found by Koponen et al. [43]. In contrast, other studies found that at low temperatures, N_2_O emissions may be hindered by soil N and water content [44], [45]. However, in different experimental sites, N_2_O emission was often related to increased soil temperature [46], [47]. These studies demonstrated that when soil moisture and N fertilization were not limiting factors to N_2_O emission, the rate of N_2_O emission increased as soil temperature increased [22].

Similarly, soil pH also influenced N_2_O production in soil (Fig. 4B). N_2_ was mainly produced through denitrification when the soil pH was neutral, and the N_2_O/N_2_ ratio increased when soil pH decreased [48]. In our study, when soil pH values decreased with irrigation, N_2_O emissions significantly increased, however, there was no relation to N_2_O emission in periods of without irrigation, so soil pH does not directly cause soil GHG emissions [36] but via affected the action of microbes [49]. On the other hand, the predominant form of nitrogen is NO_3_-N or NH_4_-N after sufficient mixed between soil and straw through tillage, which may produced little N_2_O in soil, particularly near the soil surface, with an important influence on N_2_O emissions [12].

Therefore, the CH_4_ uptake and N_2_O emissions under HTS, RTS and NTS were higher than those under HT, RT and NT, respectively, due to the effect of subsoiling. Moreover, the emission differences of CH_4_ and N_2_O between HTS, RTS and NTS were largely due to the original tillage systems, because they had different background value of soil environment factors, these soil factors change extent after conversion highly affected on CH_4_ and N_2_O emissions among treatment in this study. Therefore, the variations in CH_4_ uptake and N_2_O emissions correlated with subsoiling are mainly due to alterations in soil conditions resulting from subsoiling, including soil temperature, moisture, NH_4_^+^-N, SOC and pH.

### GWP of CH_4_ and N_2_O after Conversion to Subsoiling

Although there was a negative effect on the GWP of N_2_O after conversion to subsoiling, the increased CH_4_ absorption by soils partially counteracted this negative effect. The total GWP of CH_4_ and N_2_O increased slightly compare with the original tillage systems, especially under HTS and RTS (Table 1). Some previous studies reported that no-tillage is a better tillage system at mitigating GHG emissions [6], [50], and the lowest GWP of CH_4_ and N_2_O was only measured under NT in this study. However, the GWP of CH_4_ and N_2_O would increase if NT was converted to NTS.

### Yield Variation after Conversion to Subsoiling

In this study, the fields where the HT, RT and NT methods were previously used showed only slight improvements in wheat grain yields between two years (Table 4), possibly due to the subsoil hardpan. However, under HTS, RTS and NTS, the number of spikes, grains per ear and 1000-grain weight significantly increased, which is in agreement with other reports in which subsoiling was found to be an effective method to increase wheat production [51]–[53].

### Conclusions

Significant variations were measured in CH_4_ and N_2_O emissions after conversion to subsoiling in the North China Plain. While the uptake of CH_4_ improved greatly, N_2_O emissions also increased after subsoiling. As a result, we demonstrated that the GWP would increase if converted from minimum or no-tillage to subsoiling, especially from no-tillage. Soil temperature, moisture, SOC, NH_4_^+^-N and pH also varied and were strongly related to CH_4_ uptake and N_2_O emissions. In addition, the original tillage systems had an important effect on soil factors and GWP variations after conversion to subsoiling. Therefore, the results of our study provide evidence that conversion from rotary tillage to subsoiling (RTS) or harrow tillage to subsoiling (HTS) had a lower GWP for CH_4_ and N_2_O compared with conversion from no-tillage to subsoiling (NTS), while the grain yields under both RTS and HTS increase. Therefore, we suggest that these two rotation tillage systems be developed in this region.
